# Sulfur nanocomposites with insecticidal effect for the control of *Bactericera cockerelli*

**DOI:** 10.3762/bjnano.14.91

**Published:** 2023-11-17

**Authors:** Lany S Araujo-Yépez, Juan O Tigrero-Salas, Vicente A Delgado-Rodríguez, Vladimir A Aguirre-Yela, Josué N Villota-Méndez

**Affiliations:** 1 Departamento de Ciencias de la Vida y la Agricultura, Universidad de las Fuerzas Armadas – ESPE, Av. General Rumiñahui 171-5-231B, Sangolquí, PO Box 171-5-231B, Ecuador; 2 Centro de Nanociencia y Nanotecnología (CENCINAT), Universidad de las Fuerzas Armadas – ESPE, Av. General Rumiñahui 171-5-231B, Sangolquí, PO Box 171-5-231B, Ecuador

**Keywords:** eucalyptus, nanoinsecticide, nanotechnology, Paratrioza control, rosemary

## Abstract

The purpose of this research was to synthesize nanocomposites consisting of sulfur nanoparticles coated with eucalyptus and rosemary essential oils to determine the insecticidal effect in the control of nymphs of paratrioza (*Bactericera cockerelli* (Sulc) (Hemiptera: Triozidae)) in potato crops. A solution of thiosulfate was reduced to elemental sulfur, and the sulfur nanoparticles were coated with eucalyptus and rosemary essential oils with the three concentrations of 0.25%, 0.5%, and 0.75%. The samples were characterized by UV–visible spectroscopy, energy-dispersive X-ray spectroscopy, transmission electron microscopy, and scanning electron microscopy. The insecticidal efficacy of the nanocomposites was evaluated in the entomology laboratory 24, 48, and 72 h after application. Furthermore, efficacy was compared to the commercial insecticide thiamethoxam (0.25%) and a control. The results show that eucalyptus nanocomposites with oil concentrations of 0.25%, 0.5%, and 0.75% and rosemary nanocomposites with an oil concentration of 0.5% have an insecticidal efficacy of 100% for the control of insect nymphs 24 h after application. The insecticidal efficacy of rosemary nanocomposites with oil concentrations of 0.25% and 0.75% increases over time and reaches 100% at 24 and 72 h, respectively. The synthesized nanocomposites are more effective in controlling nymphs of paratrioza than the commercial insecticide thiamethoxam; thus, they could be used for the development of new insecticides.

## Introduction

Paratrioza (*Bactericera cockerelli* Šulc) (Hemiptera: Triozidae) is one of the most dangerous pests of potato, tomato, pepper, and other crops of the family Solanaceae [1]. The insect is one of the most destructive potato pests in the western hemisphere, New Zealand, and Australia [2]. It is native to North America. However, because of its aggressiveness, the susceptibility of cultivated species varieties, and favorable climatic conditions, it has been distributed to México, Central America, and recently to South American countries [3–5].

In Ecuador, potato cultivation is one of the main agricultural activities and generates significant income for producers and population [6]. The main problems for producers are pests and diseases that severely affect crops [7]. High densities of insects feeding on potatoes prior to flowering can result in a large number of unmarketable tubers [8]. Additionally, combined infestation with the bacterium *Candidatus Liberibacter solanacearum* causes abnormal plant development and early death, reducing the quality and yields of potato, tomato, and pepper crops [2].

*Bactericella cockerelli* tends to be difficult to manage with synthetic insecticides, such as organophosphates, organochlorines, carbamates, and pyrethroids, that are used to combat this pest [9]. The insect pest has developed resistance through high fecundity and short doubling time [10]. Also, persistence, bioaccumulation, toxicity, misuse, and overuse of synthetic insecticides have led to deterioration of soil, air pollution, contamination of water bodies, degradation of agroecosystems, and damages to human health after direct or indirect exposure [11–13]. Therefore, new methods need to be considered to control the pest. Nanotechnology has emerged as a technological advance that can enhance modern agriculture by helping in the development of new nanoinsecticides to combat pests in a more productive, cost-effective, and eco-friendly way [8,12].

Nanoscale agricultural products are developed using nanotechnology, such as nanopesticides, nanoinsecticides, nanoemulsions, and nanoparticles, to reduce the use of toxic chemicals [14]. Furthermore, different kinds of polysaccharides (e.g., chitosan, alginates, and polyethylene glycol) have been used for the synthesis of nanoinsecticides [15]. While other forms of polymer and non-polymer nanoformulations, such as nanofibers, nanocapsules, nanogels, nanomicelles, and nanospheres, have been used for the encapsulation of nanoinsecticides [16].

The technique used to improve the insecticidal efficacy is called nanoencapsulation [14]. For this purpose, nanometer-sized active ingredients are filled into a thin-walled sac, so that they can be slowly released throughout the whole process. This not only improves the yield; it also reduces the amount of required pesticide and environmental hazards [16].

Sulfur is considered one of the oldest pesticides used in agriculture for the treatment of a wide range of plant diseases [17]. Elemental sulfur in nanoparticulate forms can be generated by different chemical methods [18–19]. Elemental sulfur nanoparticles (SNPs) have already demonstrated significant insecticidal, fungicidal, and bactericidal activity [20–21]. By manipulating particle size and surface area, SNPs can exhibit higher absorption, increase the efficacy of new insecticide formulations, and reduce the amount of insecticide required for pest control [22]. Nanoparticles are known for their insecticidal properties; they interact with the cell membranes of the insects, causing the denaturation of organelles and enzymes, oxidative stress, and cell death [23–24].

Essential oils are potential botanical sources for developing new insecticides [25]. Their active components act against pest species through toxicant and repellent effects, developmental and behavioral alterations, and induction of sterility or infertility of insects [26]. Technologies such as nanoformulations or microencapsulation of essential oils protect their active components from degradation and evaporation losses, thus enhancing their stability and solubility [27].

In this framework, this research shows the synthesis of nanocomposites (NCMPs) containing elemental sulfur nanoparticles coated with essential oils of eucalyptus and rosemary at different concentrations. Characterizations were carried out through UV–visible spectroscopy, energy-dispersive X-ray spectroscopy (EDS), transmission electron microscopy (TEM), and scanning electron microscopy (SEM); also the insecticidal efficacy of the NCMPs for the control of nymphs of paratrioza was evaluated.

## Results and Discussion

### Sulfur nanoparticles

**Figure 1:**
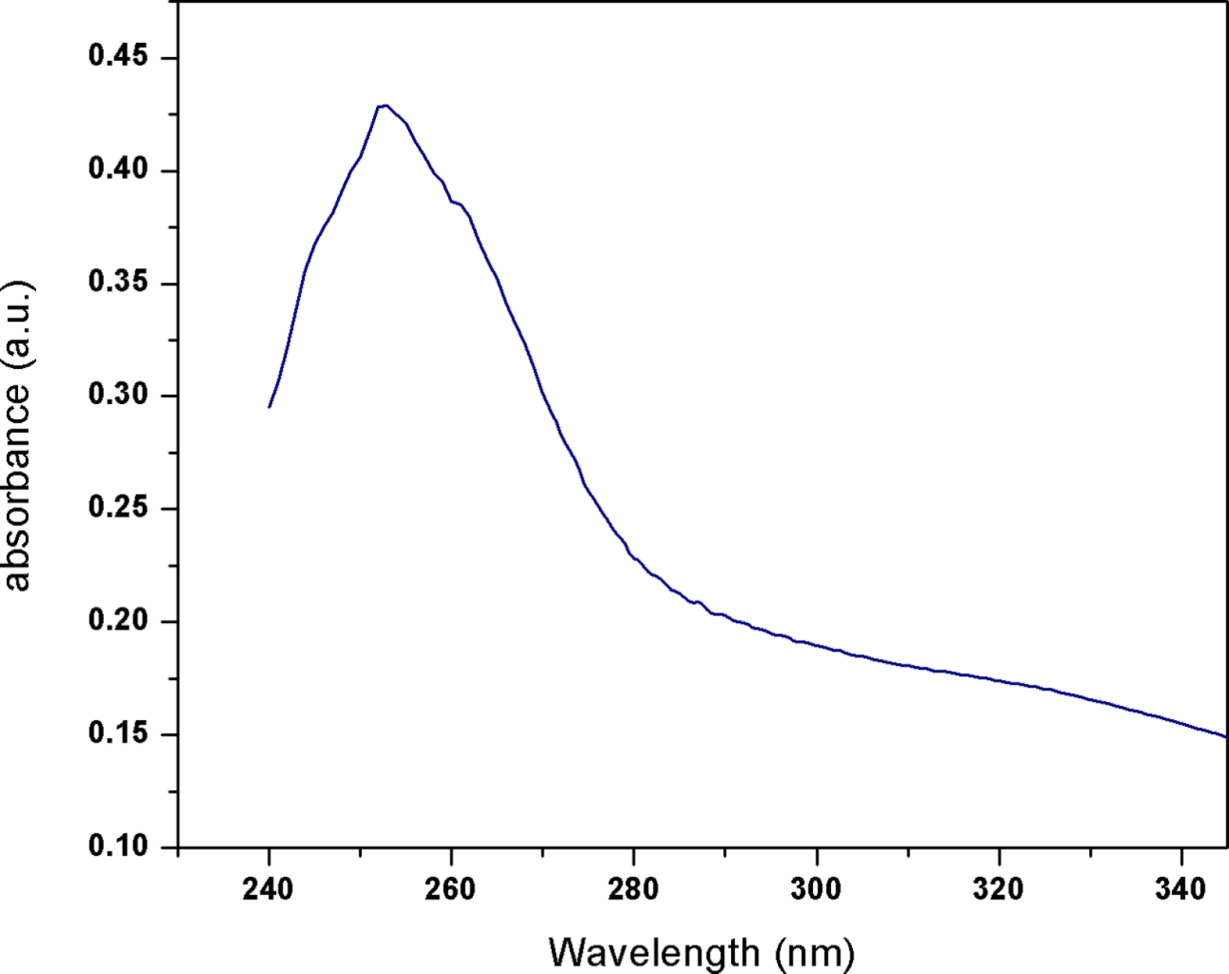
UV–vis spectrum of SNPs.

Figure 1 shows the UV–visible spectrum of the synthesized SNPs. A maximum absorption peak was observed at 253 nm. This absorption due to the n→σ* transition of nonbonding electrons is a confirmation of SNP formation [28–29]. Furthermore, it is well known that α-sulfur shows a maximum absorption peak in the range of 250 to 400 nm [30–31]. Other researchers found clear light absorption peaks of SNPs at 240, 260 and 280 nm [32–33].

**Figure 2:**
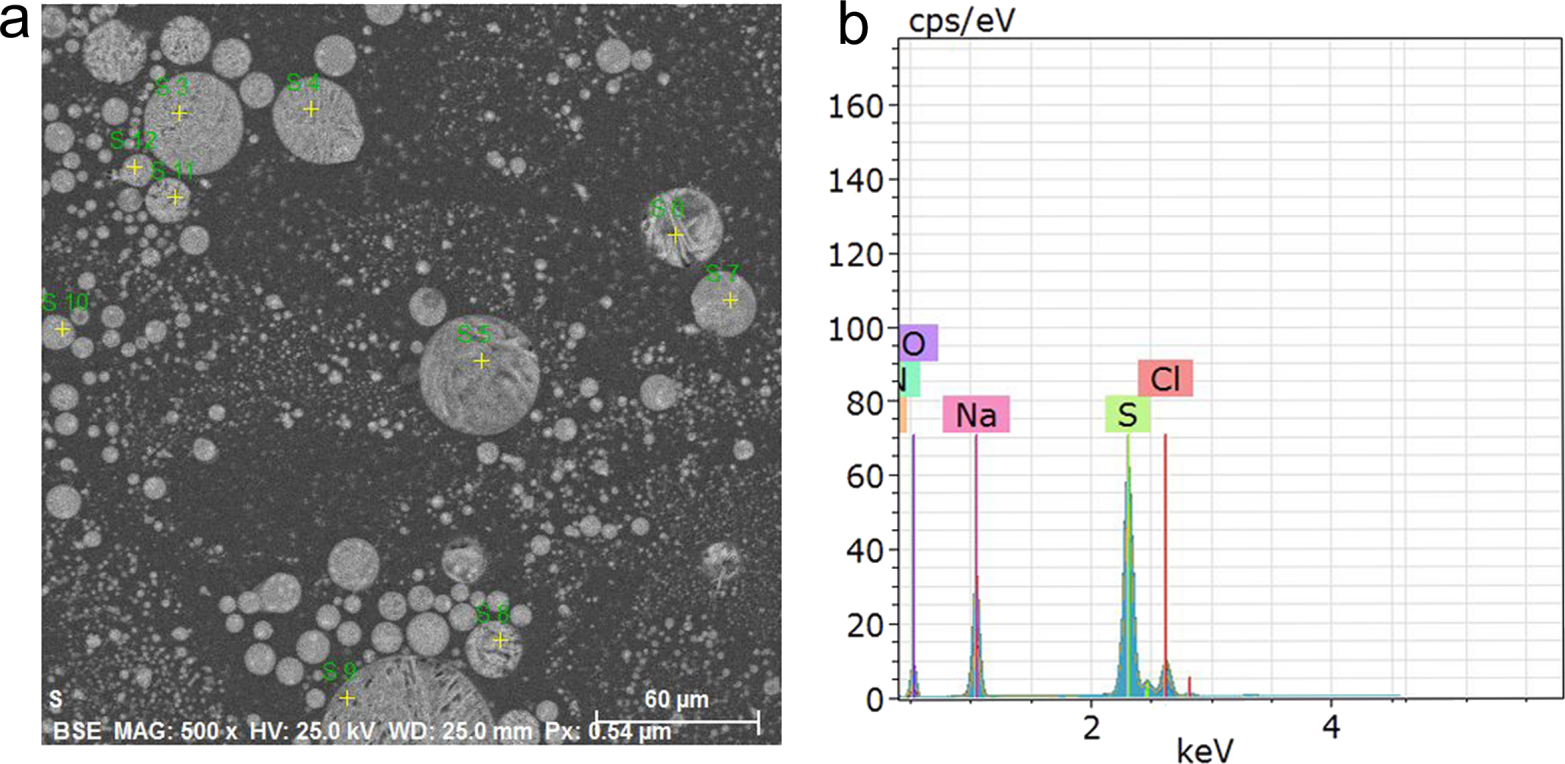
(a) SEM micrograph and (b) EDS spectrum of synthesized SNPs.

EDS analysis (Figure 2) shows the presence of sulfur at a fraction of 28 wt %. In addition, Na, Cl, and O, corresponding to the by-products of the reduction of sodium thiosulfate to sulfur (see Experimental section), were found [19,31,34]. Carbon is from the substrate used in the EDS analysis [35–36].

**Figure 3:**
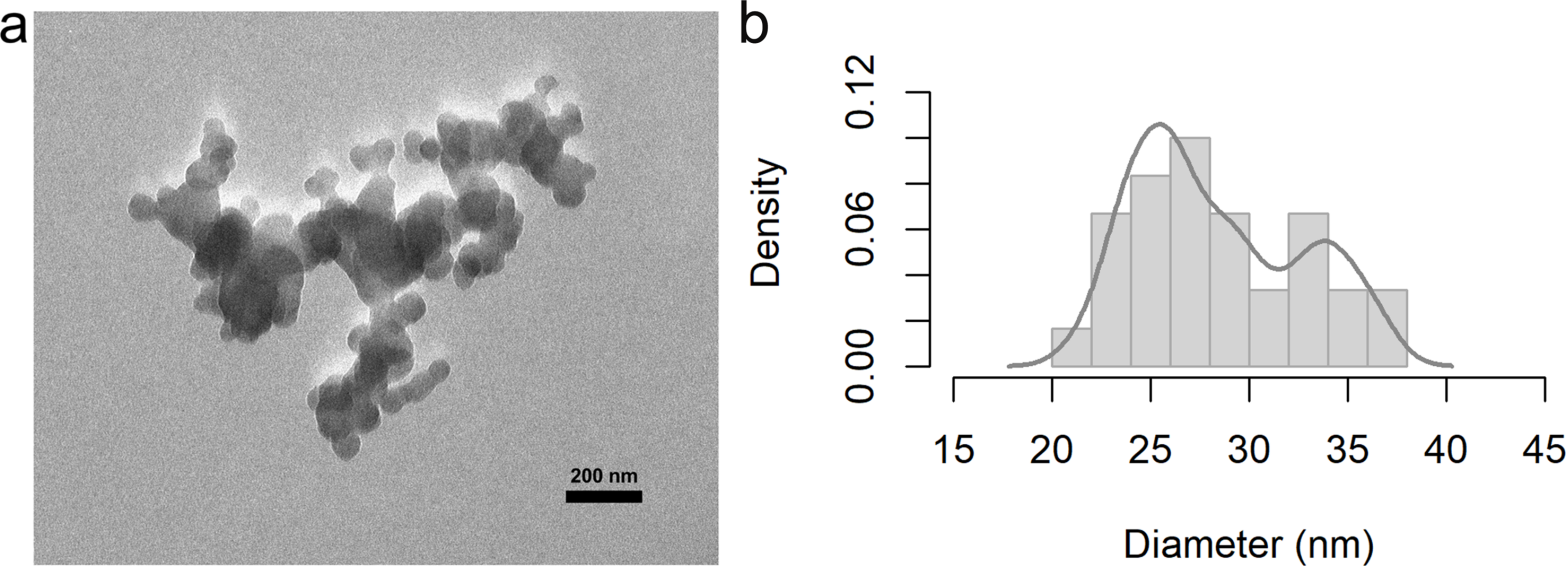
(a) TEM micrograph of synthesized SNPs and (b) density function.

The TEM micrograph in Figure 3a reveals the formation of spherical SNPs that agglomerate into larger and smaller clusters, whose diameters can be estimated from the density function in Figure 3b. The formation process appears to be influenced by two key steps, namely nucleation and growth. They are crucial in the formation of nanoparticles and control various properties of the final product, such as size, distribution, and the nature of the particles [37]. Furthermore, the observed diameters align with other research in which sizes in the range of 10–80 nm were obtained using the same method for SNP synthesis, that is, chemical precipitation from sodium thiosulfate as a sulfur source [31–32]. Those studies reported that the size of the SNPs increased with an increasing concentration ratio between acid and sulfur source during the synthesis [33–34].

### Nanocomposites

**Figure 4:**
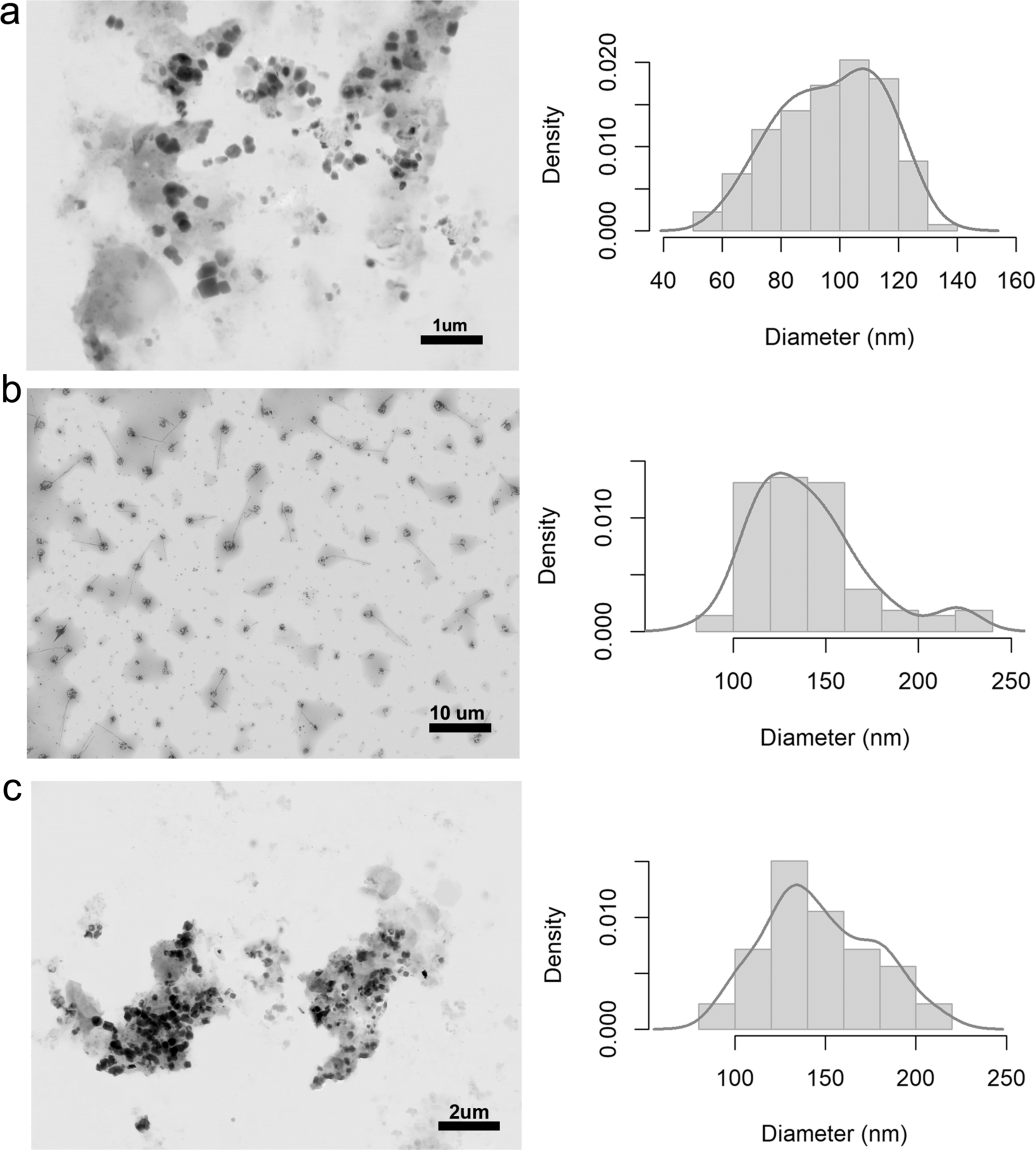
STEM micrographs (left) and density plots (right) of eucalyptus nanocomposites with oil concentrations of (a) 0.25%, (b) 0.5%, and (c) 0.75%.

**Figure 5:**
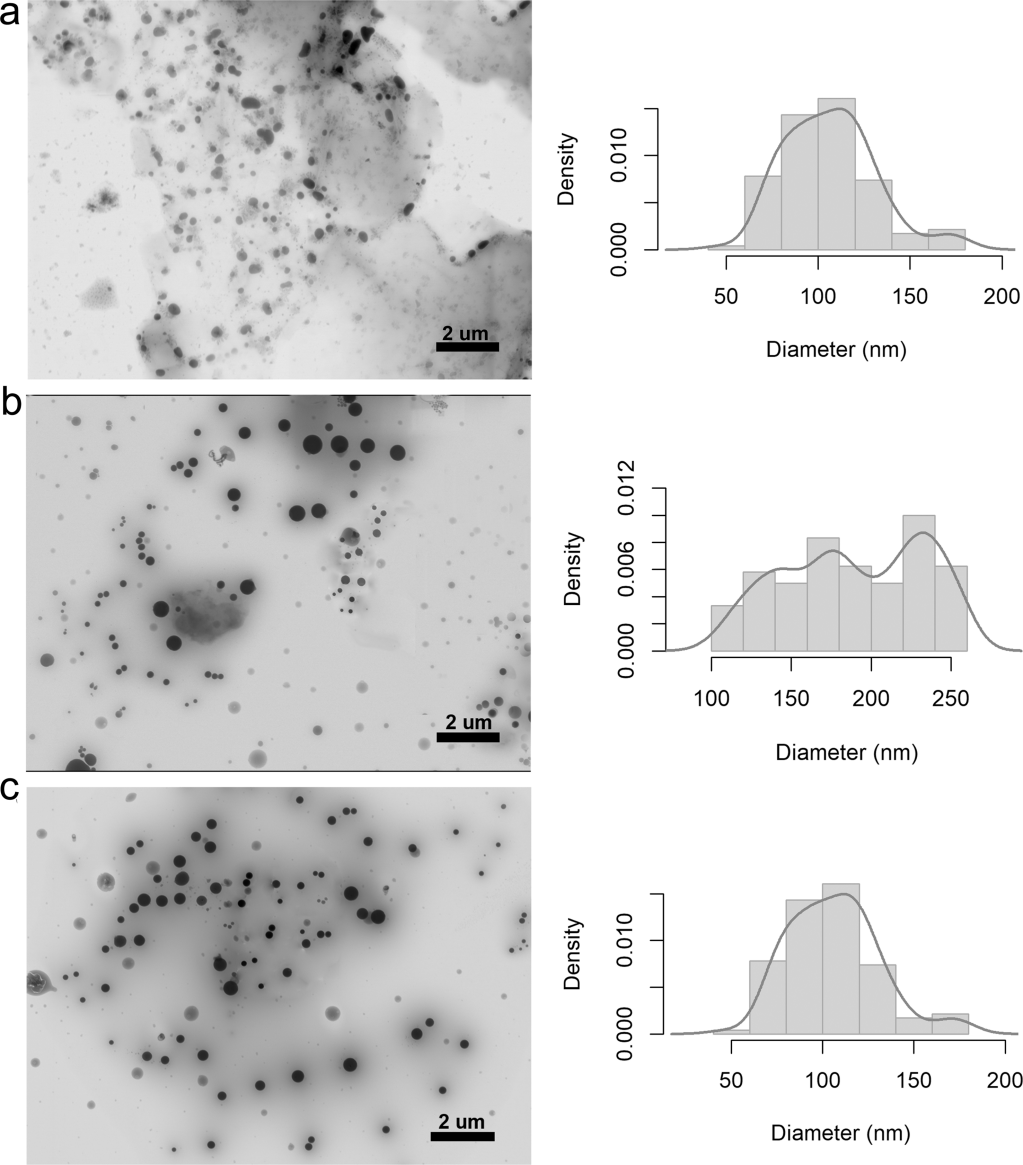
STEM micrographs (left) and density plots (right) of rosemary nanocomposites with oil concentrations of (a) 0.25%, (b) 0.5%, and (c) 0.75%.

An analysis of morphology and size using STEM revealed that the NCMPs with eucalyptus and rosemary oils had spherical shapes (Figure 4 and Figure 5). Furthermore, the diameters ranged from 57 to 136 nm, 80 to 227 nm, and 88 to 215 nm for NCMPs containing eucalyptus essential oil at concentrations of 0.25%, 0.5%, and 0.75%, respectively (Figure 4), while the particle diameters ranged from 47 to 178 nm, 101 to 258 nm, and 110 to 292 nm for nanocomposites with rosemary oil at concentrations of 0.25%, 0.5%, and 0.75%, respectively (Figure 5). The density plots and the statistics (Table 1) show particle sizes without a particular distribution, and the increase in size and accumulation of the nanocomposites may be influenced by processes that involve classical nucleation, aggregation, and/or Ostwald ripening [38].

**Table 1:** Descriptive statistics for the size distributions of eucalyptus and rosemary nanocomposites.

Treatment	Concentration (%)	Descriptive statics (nm)								

		Mean	SD	MAD	Min	Max	Range	IQR	Q0.25	Q0.75

SNPs	—	29.04	6.52	8.36	17.78	40.31	22.52	11.26	23.41	34.67

eucalyptus NCMPs	0.25	96.5	33.3	42.71	39	154	115	57.5	67.75	125.25
	0.50	135.5	63.41	81.33	44	263	219	109.5	98.75	208.25
	0.75	151.5	55.88	71.68	55	248	193	96.5	103.25	199.75

rosemary NCMPs	0.25	112.5	55.3	70.93	17	208	191	95.5	64.75	160.25
	0.50	179.5	66.3	85.04	65	294	229	114.5	122.25	236.75
	0.75	201	83.96	107.7	56	346	290	145	128.5	273.5

**Figure 6:**
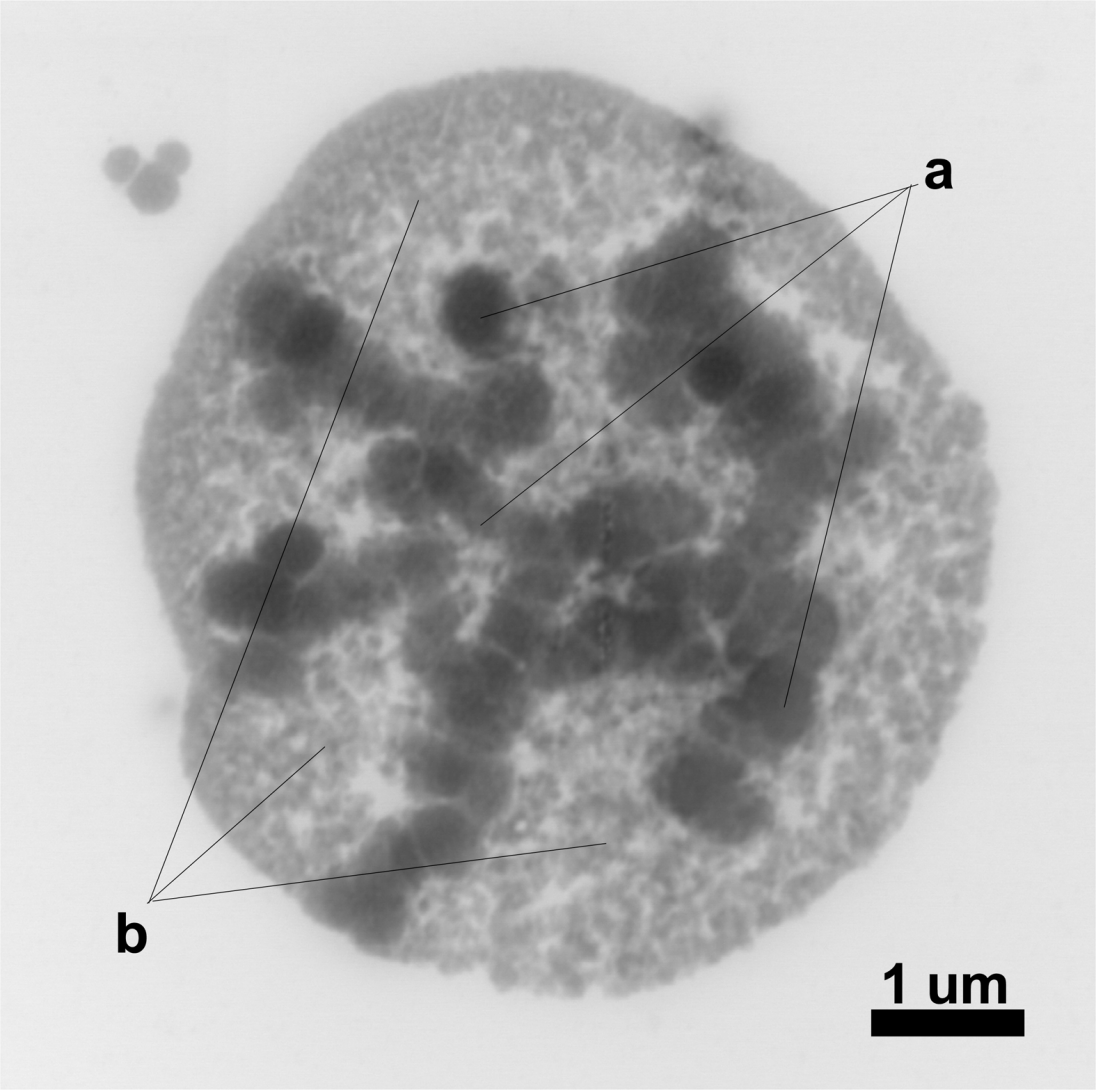
STEM micrograph of a rosemary NCMP with 0.75% oil concentration: (a) aqueous phase and (b) oily phase.

Figure 6 shows a STEM micrograph of the nanomicellar structure of a rosemary NCMP composed of two immiscible phases, that is, (a) the aqueous phase formed by the sulfur nanoparticles and (b) the oily phase formed by rosemary essential oil. Ethanol used during synthesis acts as a cosurfactant. Because of its amphiphilic properties with a hydrocarbon chain and a hydroxy group it can reduce the interfacial tension between the two immiscible phases [39–40].

The size of the NCMPs depends on the concentration of eucalyptus and rosemary essential oils. This is in agreement with similar studies where an increase in essential oil concentration influenced the viscosity of the oily phase of the nanomicelle, increasing the diameter of the particles [41–42]. A possible reason is that the viscosity of the oil will influence the rate at which cosurfactant molecules move from the oily phase to the aqueous phase. The lower the oil viscosity, the faster the cosurfactant molecules can move and the smaller the formed nanomicelles [43]. Furthermore, the diameters agree with other research that obtained *Eucalytus globulus* oil nanomicelles with sizes up to 100 nm [39]. Other authors obtained *Mentha piperita* and grape seed oil nanomicelles with sizes of 200 and 500 nm, respectively [41,44].

### Evaluation of insecticidal efficacy of nanocomposites

The different modes of insecticidal treatment and the results of these treatments are shown in Table 2 and Table 3, respectively. 24 h after application, treatments T1, T2, T3, and T5 showed an insecticidal efficacy of 100%. As time went on, the insecticidal efficacy of the other treatments increased. At 48 h, the T4 treatment reached an efficacy of 100%, and after 72 h the T6 treatment exhibited 100% efficacy. Treatment with thiamethoxam (T7) had a significantly lower insecticidal efficacy than nanocomposite treatments in the evaluated period of time. The control group (T8) showed the lowest values of insecticidal efficacy among all evaluated treatments.

**Table 2:** Abbreviations of treatments.

Abbreviation	Treatment	Concentration (%)

T1	eucalyptus NCMPs	0.25
T2		0.50
T3		0.75

T4	rosemary NCMPs	0.25
T5		0.50
T6		0.75

T7	thiamethoxam	0.25

T8	control	0.00

**Table 3:** Insecticidal efficacy of treatments with eucalyptus NCMPs, rosemary NCMPs, and thiamethoxam against *Bactericera cockerelli* paratrioza nymphs.

Treatment	Concentration (%)	Insecticidal efficacy (mean ± SD)		

		24 h	48 h	72 h

eucalyptus NCMPs	0.25	100.00 ± 3.33^abcd^	100.00 ± 4.86^abcd^	100.00 ± 1.67^a^
	0.50	100.00 ± 3.33^ab^	100.00 ± 4.86^ab^	100.00 ± 1.67^ab^
	0.75	100.00 ± 3.33^abc^	100.00 ± 4.86^abc^	100.00 ± 1.67^abc^

rosemary NCMPs	0.25	90.00 ± 3.33^bcdef^	100.00 ± 4.86^ab^	100.00 ± 1.67^abc^
	0.50	100.00 ± 3.33^abc^	100.00 ± 4.86^abcd^	100.00 ± 1.67^abc^
	0.75	96.67 ± 3.33^abcde^	96.67 ± 4.86^abcde^	100.00 ± 1.67^abc^

thiamethoxam	0.25	50.00 ± 3.33^g^	70.00 ± 4.86^h^	83.33 ± 1.67^f^

control	0.00	13.33 ± 3.33^i^	26.67 ± 4.86^j^	43.33 ± 1.67^g^

^a–j^Values with the same letters did not differ significantly.

Also, nanoencapsulation is known to improve the insecticidal efficacy because the higher surface area and specificity provide stronger contact of the active substance with the insects [45]. The working mechanism of the nanocomposites may be the effective penetration through pores and microfibrils of the insects’ cuticle [45] and the subsequent release of essential oil and sulfur nanoparticles, interfering with biology, physiology, and nervous system [46].

The use of elemental sulfur as an insecticide in the control of parasitic insect nymphs has been reported [47]. Other authors have reported the use of sulfur nanoparticles against larvae, pupae, and adults of the fruit fly *Drosophila melanogaster* [48].

In addition, nanoencapsulated essential oils have chemical activity and increased mobility, allowing for the penetration into insect tissues through the cuticle or by ingestion through the digestive tract [49]. Essential oils are lipophilic and, thus, can enter the insect and cause biochemical dysfunction and mortality [50]. Rosemary essential oil-laden nanoformulations have shown significant insecticidal activity for the effective management of the red beetle *Tribolium castaneum* [51]. Another study claimed that eucalyptus essential oil-laden nanoemulsions had insecticidal activity against *Sitophilus oryzae* in rice crops [52].

## Conclusion

Nanocomposites with a nanomicellar structure were synthesized. They are composed of an aqueous phase of elemental sulfur nanoparticles that agglomerate into clusters of smaller and larger particles and an oily phase made up of eucalyptus or rosemary essential oils with concentrations of 0.25%, 0.5%, and 0.75%. The increase in size of the nanocomposites is related to agglomeration and effects such as Ostwald ripening, or the increase in essential oil concentration. In addition, the insecticidal efficacy of the synthesized nanocomposites was evaluated. 24 h after application, eucalyptus nanocomposites with oil concentrations of 0.25%, 0.5%, and 0.75% as well as rosemary NCMPs with 0.5% oil concentration exhibited an insecticidal efficacy of 100%. The insecticidal efficacy of rosemary nanocomposites with 0.25% and 0.75% oil concentration increased over time, reaching 100% at 24 and 72 h, respectively. The treatment with thiamethoxam with 0.25% oil concentration had a significantly lower efficacy than the treatments with the nanocomposites. Hence, the synthesized nanocomposites are more effective for the control of paratrioza nymphs than the commercial insecticide thiamethoxam. The nanocomposites can be used potentially for integral pest management programs and the development of new insecticides.

## Experimental

### Materials

Sodium thiosulfate pentahydrate (Na_2_S_2_O_3_·5H_2_O, ACS reagent, ≥99.5%, CAS number: 10102-17-7), hydrochloric acid (HCl, ACS reagent, 37%, CAS number: 7647-01-0), triethanolamine ((HOCH_2_CH_2_)_3_N, ACS reagent, ≥99.5%, CAS number: 102-71-6), ethanol (CH_3_CH_2_OH, CAS number: 64-17-5), and polyethylene glycol (PEG, H(OCH_2_CH_2_)*_n_*OH, Wt: 6000, CAS number: 25322-68-3), were acquired from Sigma-Aldrich. The chemical insecticide thiamethoxam (C_8_H_10_ClN_5_O_3_S, CAS number 153719-23-4, concentration 0.25%) was acquired from Syngenta. Distilled water was obtained in the laboratory.

### Essential oil extraction

A total of 2 kg of rosemary leaves and stem and 2 kg of eucalyptus leaves were purchased in a local market in Sangolquí, Ecuador. The extraction of essential oils was carried out by steam distillation, using a Clevenger apparatus and an extractor, following the protocol described in [53–54]. The obtained oils were stored in amber glass bottles at 4 °C for later use.

### Synthesis of sulfur nanoparticles

In 25 mL of a 0.01M solution of thiosulfate pentahydrate, 250 μL of a 2 M solution of hydrochloric acid was added under stirring at 25 °C. The ratio of molar concentration of thiosulfate to HCl was 1:2 for all assays. After 30 min, the redox reaction to form sulfur reached equilibrium (Equation 1).


[1] $${\text{Na}}_{2}{\text{S}}_{2}{\text{O}}_{3}+2\text{HCl}\to 2\text{NaCl}+{\text{SO}}_{2}+{\text{S}}_{↓}+{\text{H}}_{2}\text{O}$$

### Synthesis of nanocomposites

Solutions of ethanol/essential oil were prepared using oil concentrations of 0.25%, 0.5%, and 0.75%. Subsequently, the previously synthesized sulfur nanoparticle solution was added (Table 4). Finally, 1 mL of 1% PEG was added to each solution as stabilizing agent.

**Table 4:** Amount of sulfur nanoparticles added to ethanol/essential oil solutions.

Solution of ethanol/essential oil	Added sulfur nanoparticle solution (mL)

ethanol/eucalyptus oil 0.25%	25
ethanol/eucalyptus oil 0.5%	18
ethanol/eucalyptus oil 0.75%	20
ethanol/rosemary oil 0.25%	37
ethanol/rosemary oil 0.5%	29
ethanol/rosemary oil 0.75%	26

### Characterization techniques and equipment

UV–visible spectroscopy was performed on an Analytik Jena SPECORD^®^ S 600 spectrophotometer. Size and morphology of the SNPs were determined using an FEI Tecnai G2 Spirit Twin transmission electron microscope. Energy-dispersive X-ray spectroscopy analysis of SNPs was performed on a Phenom ProX scanning electron microscope equipped with a QUANTAX-EDS detector, using a voltage of 25 kV and the Prosuite software. Size and morphology of NCMPs were determined with a Tescan MIRA3 scanning electron microscope.

### Sampling of paratrioza nymphs

For experimentation with nymphs of the paratrioza insects, sampling trials and evaluation of insecticidal efficacy were conducted in accordance with the pertinent laws and institutional guidelines of the technical cooperation agreement between the Phytosanitary Regulation and Control Agency (AGROCALIDAD) and the Universidad de las Fuerzas Armadas – ESPE.

Leaves infested with paratrioza nymphs were collected from a potato plantation located at the IASA I campus of the Universidad de las Fuerzas Armadas – ESPE. The samples were taken to the entomology laboratory under standard insectary conditions (27 ± 1 °C temperature, 80 ± 10% relative humidity, and 12 h light/12 h dark photoperiod) [55].

### Evaluation of insecticidal efficacy of nanocomposites

The evaluation was carried out in vitro in the entomology laboratory of the Universidad de las Fuerzas Armadas – ESPE with eight treatments (Table 2). The experimental setup was a 250 mL polypropylene jar containing ten nymphs of the insect placed on a potato leaf on absorbent paper moistened with distilled water. A polyester mosquito net with a mesh size of 256 was used to cover the jar opening, allowing for air circulation, and preventing other organisms from entering. The test was repeated three times and distilled water was used as control. All treatments were applied with a fine-drop sprayer. Mortality was recorded 24, 48 and 72 h after application. Nymphs were considered dead when they did not move at all after stimulation.

### Data analysis

Based on mortality data, the percentage efficacy of treatments was calculated using the Henderson–Tilton formula [56]:


[2] $$\text{effectiveness}\left[\%\right]=\frac{b-k}{100-k},$$

where *b* is the percentage of dead individuals of the treatment group and *k* is percentage of dead individuals in the control group.

Data were analyzed with InfoStat software followed by a Fisher's LSD significance test. Results were expressed as means (± SD) of data and were considered significantly different at *p* < 0.05.

The diameter probability density distribution of SNPs and NCMPs were estimated using the kernel density estimation methodology. Additionally, the descriptive statistics for each resulting distribution were calculated.
